# Associations Between Child Maltreatment and Depressive Symptoms Among Chinese College Students: An Analysis of Sex Differences

**DOI:** 10.3389/fpsyt.2021.656646

**Published:** 2021-07-09

**Authors:** Xiaoliang Chen, Sheng Zhang, Guoliang Huang, Yan Xu, Qian Li, Jingman Shi, Wenyan Li, Wanxin Wang, Lan Guo, Ciyong Lu

**Affiliations:** ^1^Department of Medical Statistics and Epidemiology, School of Public Health, Sun Yat-sen University, Guangzhou, China; ^2^Guangdong Engineering Technology Research Center of Nutrition Translation, Guangzhou, China; ^3^Center for ADR (Adverse Drug Reaction) Monitoring of Guangdong, Guangzhou, China

**Keywords:** child maltreatment, depressive symptoms, college students, cumulative effect, sex

## Abstract

**Background:** Depressive symptoms and child maltreatment are both global public health problems among young adults. This study aimed to investigate the associations between five types of child maltreatment and depressive symptoms among Chinese college students, with a focus on potential sex differences.

**Methods:** A cross-sectional study of a nationally representative sample of Chinese college students was conducted from March to June 2019 with a multistage, stratified cluster, random sampling method. In total, 30,179 college students from 60 colleges of 10 Chinese province-level regions completed standard questionnaires, including a history of child maltreatment and current depressive symptoms.

**Results:** The prevalence of depressive symptoms among college students in China was 7.3%. After adjusting for control variables, physical abuse (adjusted odds ratio [aOR] = 1.20, 95% confidence interval [CI] = 1.17–1.23), emotional abuse (aOR = 1.21, 95% CI = 1.19–1.23), sexual abuse (aOR = 1.19, 95% CI = 1.16–1.22), physical neglect (aOR = 1.14, 95% CI = 1.12–1.16) and emotional neglect (aOR = 1.08, 95% CI = 1.07–1.09) were all positively associated with depressive symptoms. Notably, a cumulative effect of child maltreatment on depressive symptoms among Chinese college students was observed. Moreover, sex differences in the associations of emotional abuse, emotional neglect, and the number of maltreatment types with depressive symptoms were statistically significant (*P* < 0.05). Further stratification analyses showed that female students who experienced emotional abuse and emotional neglect had a higher risk of depressive symptoms than male students, and the cumulative effect of maltreatment types was stronger for females than males.

**Conclusion:** Five types of child maltreatment and their co-occurrence were associated with an increased risk of depressive symptoms among college students. Furthermore, the effects of emotional abuse, emotional neglect and the number of maltreatment types on depressive symptoms were stronger for females than for males. These findings can promote understanding of the effects of child maltreatment on depressive symptoms, and prevention and intervention strategies for depressive symptoms should consider the type of child maltreatment and sex differences.

## Introduction

Depressive symptoms are a common mental health problem worldwide and are a leading contributor to the global burden of disease in young people (1). According to national surveys in the U.S., the prevalence of depressive symptoms in college students has risen in recent years (2, 3). In the transition from adolescence to adulthood, college students are vulnerable to developing depression in the context of managing enormous pressures from academic demands, interpersonal relationships, finances, and employment (4). Depressive symptoms are one of the most prevalent mental disorders among college students (5, 6). A meta-analysis including 37 studies from different countries reported an overall prevalence of depressive symptoms (24.4%) among college students (7), which was substantially higher than that of the general population. Moreover, the prevalence of depressive symptoms among Chinese college students was estimated to be 11.7% (8). Depressive symptoms at an early age could result in a series of mental health disorders later in life and physical health diseases (9), unemployment, and suicidal behavior (10, 11). Although the etiology of depression is complex and multifactorial, the vital influence of child maltreatment on depression has been generally recognized (12).

Child maltreatment is a widespread, global problem affecting millions of children worldwide (13). It takes different forms: physical abuse, emotional abuse, sexual abuse, physical neglect and emotional neglect. Under the influence of Confucianism, Chinese traditional culture emphasizes children's obedience to parental authority (14). In China, child maltreatment is common (15). Child maltreatment not only adversely affects people's social functioning, mental health, and physical health in the short and long term (16) but also leads to a heavy economic burden (15). Previous studies have reported positive associations between various types of child maltreatment and depressive symptoms (17–19). Most of these studies were conducted in Western or developed countries. For example, Canadian research indicated that a history of child physical and sexual abuse was associated with depression (20). Different types of maltreatment often co-occur (21). Exposure to multiple types of maltreatment may further elevate the risk of adverse psychological outcomes, including depressive disorders, anxiety disorders and posttraumatic stress disorders, in early adulthood, leading to cumulative damage to victims' mental health (22).

Women are approximately twice as likely to develop depression as men (23). Men and women have different prevalence rates of experiences of different types of child maltreatment, such as sexual abuse and verbal abuse (a form of emotional abuse) (13, 24). It is not clear whether the effects of child maltreatment on depressive symptoms differ by sex. Only a few studies in Western countries have investigated whether sex moderates the effects of child maltreatment on depressive symptoms, but their findings are inconsistent. Youssef et al. reported that females with a history of emotional neglect were significantly more susceptible to depressive symptoms than males (25). Roxburgh and MacArthur observed that men who reported having experienced sexual assault were more depressed than women (26). However, a study found no sex differences in the associations between all types of child maltreatment and depression in adulthood (27).

To our knowledge, there is a dearth of studies investigating the associations between single and multiple types of child maltreatment and depressive symptoms in college students, and no study in China has assessed the potential moderating effects of sex on these associations among college students. Therefore, we conducted this national cross-sectional study among Chinese college students to explore the single and multiple effects of five types of child maltreatment on depressive symptoms, as well as the moderating role of sex on these effects.

## Methods

### Study Design and Participants

Data were drawn from a school-based, cross-sectional study of a nationally representative sample of Chinese college students from March to June 2019, with a multistage, stratified cluster, random sampling method. In stage 1, we stratified the province-level regions of China into three categories based on per capita gross domestic product and then randomly selected 10 for inclusion in the study: Guangdong, Shandong, Hunan, Henan, Inner Mongolia, Heilongjiang, Yunnan, Guizhou, Xinjiang and Chongqing. In stage 2, colleges in the chosen regions were divided into undergraduate universities and vocational and technical colleges, and then three colleges were randomly selected in each stratum. A total of 60 colleges were included. In stage 3, a total of 4 majors (for 4-year or more programs) or 6 majors (for 3-year programs) were randomly selected from each college, and 1 class was randomly selected from each year of each major (because senior students participate in internships outside the college, we selected only students in years 1–3 for 4-year or more majors and students in years 1–2 for 3-year majors). In the selected classes, all available students were invited to participate in the research. Finally, 30,179 qualified questionnaires were completed, with a response rate of 97.2%. With the help of trained interviewers, one class period was scheduled for students to complete anonymous self-report questionnaires in classrooms without teachers present to reduce potential information bias.

### Measures

#### Depressive Symptoms

The Center for Epidemiologic Studies Depression Scale (CES-D) was used to measure depressive symptoms; the Chinese version has been used and validated among Chinese college students (28). In the present study, the Cronbach's alpha for the CES-D scale was 0.87. This scale contains 20 items, and each item has four response options, ranging from “rarely or none of the time” to “most or all of the time.” The CES-D has a score range of 0–60. A higher score represents more severe depressive symptoms. We used a CES-D score of ≥28 to identify students at risk for subthreshold depression, also calling having depressive symptoms (29). This cutoff score has been adopted in previous studies in China (30, 31).

#### Child Maltreatment

The Childhood Trauma Questionnaire-Short Form (CTQ-SF) (32) was used to assess a history of child maltreatment. The Chinese version of the CTQ-SF has demonstrated good reliability and validity in Chinese undergraduate students (33) and is extensively used in China (34). The CTQ-SF has 5 subscales covering 5 types of child maltreatment: physical abuse, sexual abuse, physical neglect, emotional neglect, and emotional abuse. Each subscale consists of 5 questions about experiences in childhood. The responses are rated on a 5-point scale (“never,” “rarely,” “sometimes,” “often,” and “very often”), and subscale scores range from 5 to 25. Higher subscale scores indicate more severe experiences of child maltreatment.

Additionally, the severity of each type of maltreatment was categorized as none, low, moderate, and severe (32, 35). For the present study, “moderate” to “severe” child maltreatment was defined as the presence of child maltreatment (36, 37). The cutoff scores were ≥10 for physical abuse, ≥8 for sexual abuse, ≥10 for physical neglect, ≥15 for emotional neglect, and ≥13 for emotional abuse. Each type of child maltreatment was coded as yes (1) or no (0), and then the dichotomized variables were summed to assess the number of maltreatment types experienced from 0 (experienced no maltreatment) to 5 (experienced each type of maltreatment) (36, 37).

#### Demographic Variables

Demographic variables, including age, sex (“male” = 1 and “female” = 2), year in college (from 1st to 3rd year), living arrangement, household socioeconomic status (HSS), academic pressure, relationships with classmates, relationships with teachers, current smoking and current alcohol use, were collected. Sex was assessed based on students' biological sex. Living arrangement was measured by asking students which individuals mainly lived in their primary home before they turned 16 (“living with both parents” = 1, “living with a single parent” = 2, and “living with others” = 3). HSS was assessed by students' perceived level of their family financial situation (“excellent or very good” = 1, “good” = 2, and “fair or poor” = 3). Academic pressure was estimated based on students' own perception of their academic stress (“above average” = 1, “average” = 2, and “below average” = 3). Relationships with classmates/teachers were evaluated by asking students' opinion of their relations with classmates/teachers (“good” = 1, “average” = 2, and “poor” = 3). Current smoking/alcohol use was defined as having smoked/drunk in the past 30 days (“no” = 0 and “yes” = 1).

### Statistical Analysis

First, descriptive statistics were calculated and *t*-tests or chi-square tests were performed to describe the differences in depressive symptoms by demographic characteristics. Continuous data were reported as the means and standard deviations (SDs) and analyzed by *t*-tests; categorical data were reported as frequencies and percentages and analyzed by chi-square tests. The demographic variables that had *P* < 0.10 or have been widely reported in the literature (i.e., age, sex) were entered into the following multivariate logistic regression models as covariates. Covariates for adjustment included age, sex, living arrangement, household socioeconomic status, academic pressure, relationships with classmates, relationships with teachers, current smoking, and current alcohol use. Second, univariate and multivariate logistic regression models were performed to assess the associations between five types of child maltreatment and depressive symptoms, and the odds ratios (ORs) and 95% confidence intervals (95% CIs) were calculated. The associations between the number of maltreatment types experienced and depressive symptoms were also evaluated by univariate and multivariate logistic regression models. Third, to investigate whether sex moderated the association between child maltreatment and depressive symptoms, we examined multiplicative interactions by adding the product terms to the multivariate logistic regression and computing *P*-values for the interactions. If the interactions were significantly associated with depressive symptoms, further stratification analyses by sex were conducted to explore whether the effects of child maltreatment on depressive symptoms differed in males and females. Nagelkerke pseudo *R*^2^ and omnibus test of model coefficients were employed to assess the overall fit of the models. All statistical analyses were conducted using SPSS version 25 (IBM, Armonk, New York, USA). All tests were two-tailed, with statistical significance set at *P* < 0.05.

## Results

### Demographic Characteristics and Their Associations With Depressive Symptoms

The sample characteristics are presented in Table 1. Of the 30,179 students, 42.1% were male, and 57.9% were female. The participants' ages ranged from 15 to 24 years old, and the mean age was 19.9 (SD: 1.3) years. The mean CES-D score of students was 13.9 (SD: 8.6) points, and 2,193 students (7.3%) reported having depressive symptoms. The mean CTQ-SF scores for physical abuse, emotional abuse, sexual abuse, physical neglect and emotional neglect were 5.5 (SD: 1.4), 6.3 (SD: 2.3), 5.3 (SD: 1.2), 6.9 (SD: 2.6), and 7.3 (SD: 4.4), respectively. The proportions of students who reported that they had suffered “moderate” to “severe” physical abuse, emotional abuse, sexual abuse, physical neglect and emotional neglect were 2.4, 2.7, 3.0, 14.9, and 7.5%, respectively. Moreover, the groups with and without depressive symptoms differed significantly in age, living arrangement, HSS, academic pressure, relationships with classmates, relationships with teachers, current smoking, and current alcohol use and each type of child maltreatment.

**Table 1:** Demographic characteristics and their associations with depressive symptoms (*N* = 30,179).

**Variable**	**Total**	**Depressive symptoms**			
		**No**	**Yes**	**χ^**2**^/*t***	***P*-value^b^**
	**No. (%)**	**No. (%)**	**No. (%)**		
Total	30,179	27,638 (92.7)	2,193 (7.3)		
Age (years)^a^	19.9 ± 1.3	19.9 ± 1.3	19.8 ± 1.4	2.228	0.026
**Sex**					
Male	12,688 (42.1)	11,750 (92.6)	938 (7.4)	0.079	0.778
Female	17,449 (57.9)	16,174 (92.7)	1,275 (7.3)		
Missing data	42				
**Year in college**					
1st	14,579 (48.3)	13,505 (92.6)	1,074 (7.4)	4.005	0.135
2nd	10,685 (35.4)	9,933 (93.0)	752 (7.0)		
3rd	4,915 (16.3)	4,525 (91.7)	390 (8.3)		
**Living arrangement**					
Living with both parents	17,515 (58.2)	16,423 (93.8)	1,092 (6.2)	80.147	<0.001
Living with a single parent	2,483 (8.2)	2,236 (90.1)	247 (9.9)		
Living with others	10,109 (33.6)	9,239 (91.4)	870 (8.6)		
Missing data	72				
**Household socioeconomic status**					
Excellent or very good	3,122 (10.5)	2,944 (94.3)	178 (5.7)	139.315	<0.001
Good	16,423 (55.1)	15,422 (93.9)	1,001 (6.1)		
Fair or poor	10,276 (34.4)	9,272 (90.2)	1,004 (9.8)		
Missing data	358				
**Academic pressure**					
Below average	8,864 (29.5)	8,514 (96.1)	350 (3.9)	898.880	<0.001
Average	12,807 (42.6)	12,169 (95.0)	638 (5.0)		
Above average	8,365 (27.9)	7,145 (85.4)	1,220 (14.6)		
Missing data	143				
**Relationships with classmates**					
Good	22,604 (75.0)	21,498 (95.1)	1,106 (4.9)	1146.307	<0.001
Average	7,281 (24.2)	6,284 (86.3)	997 (13.7)		
Poor	235 (0.8)	127 (54.0)	108 (46.0)		
Missing data	59				
**Relationships with teachers**					
Good	17,665 (58.7)	16,846 (95.4)	819 (4.6)	772.645	<0.001
Average	12,065 (40.1)	10,801 (89.5)	1,264 (10.5)		
Poor	358 (1.2)	232 (64.8)	126 (35.2)		
Missing data	91				
**Current smoking**					
No	25,282 (83.8)	23,573 (93.2)	1,709 (6.8)	77.864	<0.001
Yes	4,897 (16.2)	4,390 (89.6)	507 (10.4)		
**Current alcohol use**					
No	20,201 (66.9)	18,893 (93.5)	1,308 (6.5)	67.648	<0.001
Yes	9,978 (33.1)	9,070 (90.9)	908 (9.1)		
CTQ-SF scores for physical abuse^a^	5.5 ± 1.4	5.4 ± 1.3	6.2 ± 2.4	−24.873	<0.001
CTQ-SF scores for emotional abuse^a^	6.3 ± 2.3	6.2 ± 2.1	8.1 ± 3.7	−38.382	<0.001
CTQ-SF scores for sexual abuse^a^	5.3 ± 1.2	5.2 ± 1.0	5.7 ± 2.1	−20.082	<0.001
CTQ-SF scores for physical neglect^a^	6.9 ± 2.6	6.8 ± 2.5	8.2 ± 3.4	−24.068	<0.001
CTQ-SF scores for emotional neglect^a^	7.3 ± 4.4	7.2 ± 4.2	9.5 ± 5.6	−24.480	<0.001

a *Data are presented as the mean ± standard deviation*.

b *T-tests were used for continuous variables; chi-square tests were used for categorical variables*.

*CTQ-SF, Childhood Trauma Questionnaire-Short Form*.

### Associations Between Child Maltreatment and Depressive Symptoms

The univariable logistic regression models showed that all types of child maltreatment were positively associated with depressive symptoms (*P* < 0.001). In the multivariable logistic regression models with adjustment for control variables, physical abuse (score increase of 1; aOR = 1.20, 95% CI = 1.17–1.23), emotional abuse (score increase of 1; aOR = 1.21, 95% CI = 1.19–1.23), sexual abuse (score increase of 1; aOR = 1.19, 95% CI = 1.16–1.22), physical neglect (score increase of 1; aOR = 1.14, 95% CI = 1.12–1.16) and emotional neglect (score increase of 1; aOR = 1.08, 95% CI = 1.07–1.09) were associated with an increased risk of depressive symptoms. The Nagelkerke pseudo *R*^2^ values indicated that the multivariable logistic regression models explained 14.8–18.6% of the variance in depressive symptoms (Table 2).

**Table 2:** Associations between child maltreatment and depressive symptoms.

**Variables**	**Depressive symptoms**			
	**cOR (95% CI)**	***P*-value**	**aOR (95% CI)^a^**	***P*-value**
Physical abuse (score increase of 1)	1.24 (1.22–1.27)	<0.001	1.20 (1.17–1.23)	<0.001
Emotional abuse (score increase of 1)	1.23 (1.21–1.25)	<0.001	1.21 (1.19–1.23)	<0.001
Sexual abuse (score increase of 1)	1.22 (1.19–1.25)	<0.001	1.19 (1.16–1.22)	<0.001
Physical neglect (score increase of 1)	1.17 (1.16–1.19)	<0.001	1.14 (1.12–1.16)	<0.001
Emotional neglect (score increase of 1)	1.09 (1.08–1.10)	<0.001	1.08 (1.07–1.09)	<0.001

a *Adjusted for age, sex, living arrangement, household socioeconomic status, academic pressure, relationships with classmates, relationships with teachers, current smoking, and current alcohol use. The Nagelkerke R^2^ values ranged from 0.148 to 0.186*.

*The omnibus tests of model coefficients were statistically significant in all the models (P < 0.001)*.

*cOR, crude odds ratio; aOR, adjusted odds ratio; CI, confidence interval*.

Concerning the cumulative effect of child maltreatment, multivariable logistic regression showed that experiences of 1, 2, 3, 4, and 5 types of maltreatment were significantly associated with depressive symptoms, with aORs (95% CIs) of 2.11 (1.87–2.39), 2.88 (2.47–3.35), 6.90 (5.27–9.03), 7.71 (5.40–11.03), and 10.52 (5.93–18.66), respectively (Table 3).

**Table 3:** Crude and adjusted odds ratios and 95% confidence intervals for cumulative child maltreatment and depressive symptoms.

**Number of maltreatment types experienced**	**Total (%)**	**cOR (95% CI)**	***P*-value**	**aOR (95% CI)^a^**	***P*-value**
0	80.6	1		1	
1	11.5	2.50 (2.23–2.81)	<0.001	2.11 (1.87–2.39)	<0.001
2	6.0	3.41 (2.97–3.91)	<0.001	2.88 (2.47–3.35)	<0.001
3	1.1	8.69 (6.85–11.04)	<0.001	6.90 (5.27–9.03)	<0.001
4	0.6	8.71 (6.37–11.93)	<0.001	7.71 (5.40–11.03)	<0.001
5	0.2	11.17 (6.99–17.84)	<0.001	10.52 (5.93–18.66)	<0.001

a *Adjusted for age, sex, living arrangements, household socioeconomic status, academic pressure, relationships with classmates, relationships with teachers, current smoking status, and current alcohol use. The Nagelkerke R^2^ value was 0.176*.

*The omnibus test of model coefficients was statistically significant in the model (P < 0.001)*.

*cOR, crude odds ratio; aOR, adjusted odds ratio; CI, confidence interval*.

### Interaction Effects Between Child Maltreatment and Sex on Depressive Symptoms

As shown in Table 4, adjusting for confounding variables, we found significant interaction effects of emotional abuse and emotional neglect with sex on depressive symptoms (*P* < 0.01). However, the adjusted interaction effects of physical abuse, sexual abuse, physical neglect with sex were not significant (*P* > 0.05). Furthermore, the effects of the number of maltreatment types experienced on depressive symptoms were significantly different between males and females (*P* < 0.01).

**Table 4:** Interaction effects between child maltreatment and sex on depressive symptoms.

**Interaction item**	***P*-value**
Physical abuse * sex	0.397
Emotional abuse * sex	<0.001
Sexual abuse * sex	0.117
Physical neglect * sex	0.088
Emotional neglect * sex	0.002
Number of maltreatment types experienced * sex	0.009

*All the models were adjusted for age, sex, living arrangements, household socioeconomic status, academic pressure, relationships with classmates, relationships with teachers, current smoking status, and current alcohol use. The Nagelkerke R^2^ values ranged from 0.148 to 0.187*.

*The omnibus tests of model coefficients were statistically significant in all the models (P <0.001)*.

### Associations Between Child Maltreatment and Depressive Symptoms, Stratified by Sex

The stratification analyses by sex are shown in Table 5. After we controlled for other variables, both emotional abuse (score increase of 1; aOR = 1.24, 95% CI = 1.21–1.26) and emotional neglect (score increase of 1; aOR = 1.09, 95% CI = 1.08–1.10) were positively associated with depressive symptoms among female students. Additionally, emotional abuse (score increase of 1; aOR = 1.18, 95% CI = 1.16–1.20) and emotional neglect (score increase of 1; aOR = 1.06, 95% CI = 1.05–1.08) were positively associated with depressive symptoms among male students. Female students exposed to emotional abuse and neglect had a higher risk of depressive symptoms than male students. Moreover, the association between the number of maltreatment types and depressive symptoms was significantly higher among females (number increase of 1; aOR = 1.83, 95% CI = 1.71–1.96) than males (number increase of 1; aOR = 1.64, 95% CI = 1.53–1.75).

**Table 5:** Associations between child maltreatment and depressive symptoms, stratified by sex.

**Variables**	**Male**		**Female**	
	**aOR (95% CI)^a^**	***P*-value**	**aOR (95% CI)^a^**	***P*-value**
Physical abuse (score increase of 1)	1.19 (1.16–1.23)	<0.001	1.20 (1.16–1.24)	<0.001
Emotional abuse (score increase of 1)	1.18 (1.16–1.20)	<0.001	1.24 (1.21–1.26)	<0.001
Sexual abuse (score increase of 1)	1.17 (1.13–1.21)	<0.001	1.20 (1.16–1.25)	<0.001
Physical neglect (score increase of 1)	1.13 (1.10–1.15)	<0.001	1.15 (1.13–1.18)	<0.001
Emotional neglect (score increase of 1)	1.06 (1.05–1.08)	<0.001	1.09 (1.08–1.10)	<0.001
Number of maltreatment types experienced (number increase of 1)	1.64 (1.53–1.75)	<0.001	1.83 (1.71–1.96)	<0.001

a *Adjusted for age, living arrangements, household socioeconomic status, academic pressure, relationships with classmates, relationships with teachers, current smoking status, and current alcohol use. The Nagelkerke R^2^ values ranged from 0.147 to 0.202*.

*The omnibus tests of model coefficients were statistically significant in all the models (P < 0.001)*.

*aOR, adjusted odds ratio; CI, confidence interval*.

## Discussion

We found that ~7.3% of college students had depressive symptoms; this prevalence is lower than that in a Chinese study among undergraduate and postgraduate students, which reported a prevalence of depressive symptoms of 11.7% (8). The difference may be due to different study populations, measurement tools, and appraisal criteria.

Moreover, we observed that 11.5% of college students experienced a single type of child maltreatment, and 7.6% reported more than one type. Similar levels of multitype child maltreatment were found in a previous study (2015 School-Based Chinese Adolescents Health Survey) (38). The co-occurrence of different types of child maltreatment is relatively common in China. In addition, our results demonstrated the cumulative effects of child maltreatment on depressive symptoms among college students, with the aOR increasing from 2.11 to 2.88, 6.90, 7.71, and 10.52 as the number of maltreatment types increased from 1 to 2, 3, 4, and 5, respectively. This finding is consistent with previous studies reporting a dose-response relationship of cumulative child maltreatment with depressive symptoms (39, 40). Previous studies suggested that an increase in the number of maltreatment types experienced was linearly associated with an increased risk of depressive symptoms (41, 42). It's worth noting that students with three or more types of maltreatment had a markedly higher risk of depressive symptoms than those with one or two types in our study. Similarly, a study found that participants with four or more types of maltreatment had more serious depressive symptoms than those with three or fewer types (43). The relationship of cumulative child maltreatment to depressive symptoms may be more complex than a linear association. The results highlight the importance of considering multiple types of maltreatment in relation to depressive symptoms. Clinical practices and intervention strategies for depressive symptoms should screen and assess different types of child maltreatment (44), and college students with three or more types of child maltreatment may need more monitoring and targeted measures in China.

After adjusting for control variables, we found positive associations of physical abuse, emotional abuse, sexual abuse, physical neglect and emotional neglect with depressive symptoms among college students. This finding is consistent with previous studies (17–19, 45). For example, Yen et al. indicated that a history of child physical abuse was significantly associated with depression in adolescents (45). Child maltreatment is likely to increase individuals' susceptibility to developing depression in adulthood when they face stressful life events (46). One possible mechanism of this effect is that child maltreatment likely results in lasting alterations in the hypothalamic–pituitary–adrenal (HPA) axis, which is responsible for the stress response (47). Specifically, early stressors, such as child abuse, can aggravate the stress-induced glucocorticoid response and reduce the expression of glucocorticoid receptors (48, 49). A growing body of clinical studies has linked child abuse to depression via changes in HPA axis function and behavior (50, 51). In addition, various biological alterations may be involved in this relationship, including neurotransmitter systems, inflammatory reactions, and brain regions relevant to mood regulation (52). Our results underscore the significant relationships between child maltreatment and depressive symptoms. However, compared with Western countries, the availability of child protectives services is still very limited in Chinese society. Effective preventive and intervention measures should be established as soon as possible in China, such as the training of professionals (53). As one of the most effective prevention approaches, the expressway noise barrier is a primary measure to reduce traffic noise. Chinese traditional Confucianism culture emphasizes children's obedience to parental authority (14), and strict parenting is common and socially accepted (54). More school-based or community-based lectures or activities about the hazards of child maltreatment and the knowledge of nurturing children are needed to raise parents' concerns for children's physical and mental health.

We investigated whether sex plays a moderating role in the relationships of the five types of maltreatment and the cumulative effects of child maltreatment with depressive symptoms. There were three significant sex differences (in emotional abuse, emotional neglect and number of maltreatment types) in the associations between child maltreatment and depressive symptoms. Further stratification analyses by sex showed that female students who reported a history of emotional abuse or emotional neglect had a significantly higher risk of depressive symptoms than male students and that the cumulative effect of maltreatment types was significantly greater for women than men. Similarly, Gallo et al. reported that the effect of emotional abuse on depressive disorder was larger for females than for males (55). Moreover, Youssef et al. observed that women exposed to emotional neglect were significantly more likely to have depressive symptoms than men (25). One possible underlying mechanism of this finding is that females who are the victims of emotional maltreatment may be predisposed to develop a negative cognitive style, which may cause female victims to be more vulnerable to depression (55). Rose and Abramson's theory indicated that child emotional maltreatment, such as insulting words, was more likely to contribute to a negative cognitive style than physical and sexual maltreatment because emotional abusers directly instilled depressive cognitions in children (56, 57). Furthermore, owing to the sex differences in HPA axis regulation, maltreated women showed a pattern of neuroendocrine hyporeactivity compared to men who experienced similar degrees of maltreatment, which may be related to different depression prevalence (58). This neurobiological mechanism may indicate why females who reported emotional abuse, emotional neglect, or multiple maltreatment had a higher risk of depressive symptoms. In our study, the magnitude of the effect of sexual abuse on depressive symptoms was greater for females than males in stratification analysis. Differently, a study in the United States found that men who experienced sexual abuse were significantly more depressed than women (26), and a systematic review indicated that the magnitude of the effect of sexual abuse on depressive symptoms was greater for men than women (59). The inconsistency may stem from cultural differences. Oriental culture is relatively conservative (60), and Chinese traditional culture emphasizes female chastity (61). Females victimized by child sexual abuse may internalize the social stigmatization of “victim of sexual abuse” and suffer from a burning sense of shame and self-denial (62). Besides, some female participants in our study may be reluctant to reveal their experiences of sexual abuse, which may lead to an underestimation of the association between sexual abuse and depressive symptoms among females. Sex-specific preventions and interventions may be recommended to better understand the potential sex differences between different types of child maltreatment and depressive symptoms.

Several limitations should be noted in our study. First, the cross-sectional research design limited our capacity to make causal inferences regarding the observed associations. Second, our study used a retrospective self-report method to collect the data, possibly introducing recall and reporting bias. Third, the sample was restricted to school students, excluding drop-outs and students who were absent on the day that the survey was administered. Child maltreatment or depressive symptoms may be more common among students who have dropped out or are absent from school. Fourth, senior students who were facing employment pressure were not included in our study, so the prevalence of depressive symptoms may have been underrated. Fifth, the pseudo *R*^2^ values of the models were small in this study. More factors (e.g., parental depression, history of bullying and substance abuse) are needed to be collected in future studies to better explain the variance in depressive symptoms (10, 12). Sixth, ~2.3% of college students were below the age of 18 in our study, and written informed consent was provided by one of their legal guardians. Participants whose parents provided consent given may not truly report their experiences of child maltreatment, introducing potential reporting bias. Besides, the investigator would emphasize to participants the anonymity of the questionnaire at the beginning of the survey to control the potential reporting bias. Despite these weaknesses, to our knowledge, this study is the first large-scale study to investigate the associations between history of child maltreatment and depressive symptoms among Chinese college students, with consideration of sex differences.

## Conclusions

In conclusion, five specific types of child maltreatment were associated with a greater risk of depressive symptoms among college students. A cumulative effect of various types of child maltreatment was found, indicating that individuals with experiences of three or more types of maltreatment have an obviously higher risk of depressive symptoms than those with fewer or no maltreatment experiences. Moreover, the effects of emotional abuse/neglect, and the cumulative effects of different types of child maltreatment on depressive symptoms are stronger in females than males. Further research is needed to confirm and extend these findings and explore the underlying mechanism.

## Data Availability Statement

The original contributions presented in the study are included in the article/supplementary material, further inquiries can be directed to the corresponding author/s.

## Ethics Statement

The studies involving human participants were reviewed and approved by the Institutional Review Board of Sun Yat-sen University, School of Public Health. Written informed consent to participate in this study was provided by the participants' legal guardian/next of kin.

## Author Contributions

CL and LG designed the study and critically revised this manuscript. XC managed the literature searches, summaries of previous related work, and wrote the first draft of the manuscript. GH, YX, XC, SZ, QL, JS, WL, and WW carried out the field research. XC and SZ undertook the statistical analysis. All authors reviewed the manuscript.

## Conflict of Interest

The authors declare that the research was conducted in the absence of any commercial or financial relationships that could be construed as a potential conflict of interest.
